# Psychological factors in functional hypothalamic amenorrhea: A systematic review and meta-analysis

**DOI:** 10.3389/fendo.2023.981491

**Published:** 2023-01-27

**Authors:** Federica Bonazza, Giuliana Politi, Daniela Leone, Elena Vegni, Lidia Borghi

**Affiliations:** ^1^ Department of Health Sciences, University of Milan, Milan, Italy; ^2^ Azienda Socio-Sanitaria Territoriale (ASST) Santi Paolo e Carlo, San Paolo University Hospital, Milan, Italy

**Keywords:** functional hypothalamic amenorrhea, amenorrhea, psychological factors, depression, eating attitudes, meta-analysis, systematic review

## Abstract

**Background:**

Psychological factors have been found to be associated with functional hypothalamic amenorrhea (FHA); however, their role in the onset or persistence of FHA is still understudied. The study aims to assess the associations of psychological factors with the presence vs the absence of FHA.

**Methods:**

A systematic literature search has been conducted across the major databases (PubMed, PsycINFO, Scopus, and Embase) to explore the psychological factors associated with FHA. The search was limited to English-written articles published from 2000 onwards. Articles were selected based on stringent inclusion/exclusion criteria. After data extraction, meta-analysis and meta-synthesis were conducted.

**Results:**

Of 349 retrieved articles, eight studies were included. Findings indicate that the main psychological factors associated to FHA seem to be depression and eating attitudes, especially drive for thinness. FHA women present higher levels of anxiety, sleep disorders, dysfunctional attitudes, and alexithymia. The meta-analysis on drive for thinness revealed that the pooled MD across the studies was statistically significant both in the fixed 0.63 (95% CI: 0.31–0.95) and random model 0.70 (95% CI: 0.13–1.26). Likewise, as for depression, the pooled MD across the studies was statistically significant both in the fixed 0.60 (95% CI: 0.36–0.84) and random model 0.61 (95% CI: 0.20–1.01).

**Discussion:**

Findings showed the association of psychological factors and FHA and recognized their involvement in the persistence of the disorder. A multidisciplinary approach should involve a collaborative process between gynecologists, clinical psychologists, and psychiatrists, from diagnosis to treatment. Longitudinal studies should be implemented with a comparison/control group or by including clinical psychologists in the psychological assessment and study design.

## 1 Introduction

Functional hypothalamic amenorrhea (FHA) is a form of secondary amenorrhea caused by hypogonadotropic hypogonadism related to an aberration of the pulsatile release of gonadotropin-releasing hormone (GnRH) from the hypothalamus (1). FHA entails a significant impact on ovarian function with hypoestrogenism and the sequent absence of a regular menstrual period persisting for more than 3-6 months in women who previously had regular cycles (2, 3).

According to epidemiological data, in Europe and USA FHA accounts for 20-35% of cases of secondary amenorrhea, rising to be one of the most common reproductive disorders in women of childbearing age (4). Among adolescent girls, the prevalence of FHA is approximately 15-48% of secondary amenorrhea diagnoses (5), but that may be underestimated due to the difficulty in differentiating from the instability of hypothalamic–pituitary-ovarian (HPO) axis during puberty (6). However, once the menstrual pattern is assumed, the approach for the diagnosis of amenorrhea does not differ from that of adults (1, 6).

FHA has been found to be related to the suppression of the HPO axis; these dysregulations of the HPO axis in FHA seemed not to be caused by any identifiable organic disease or anatomic factor (1), while they were found to be associated with stress, weight loss, and/or excessive physical exercise (7). Based on these eliciting factors, three variants of FHA have been established: weight loss-related, exercise-related, and stress-related (3). As the cause of FHA does not seem organic but functional, the role of psychological factors can be decisive in assessing their impact on the onset and persistence of the disorder. In fact, a psychogenic component has been recognized in FHA since its first diagnostic formulation (8).

Considering weight loss-related FHA, evidence shows that psychological factors interact with significant physiological changes, metabolic alterations, and endocrinological aberrations, contributing to the persistence of FHA (9, 10). Indeed, women with FHA reported more deranged eating attitudes, restrictive eating behavior, and bulimic symptoms than comparison (11). As previous studies have shown, women with dysfunctional eating attitudes are at higher risk for menstrual problems and infertility (12). As it is widely known, decreased food intake causes a shortage of energy, which leads the body to economize and thus suspend those functions not necessary for survival (such as menstruation) (13). All these features are also present more severely in patients with an eating disorder, so it is essential to make an appropriate differential diagnosis.

Likewise, exercise-related amenorrhea is a frequent clinical condition among athletes, particularly those involved in elite sports and aesthetic disciplines (1), such as artistic skating and gymnastics. Several psychological and behavioral factors have been identified as contributing to the high prevalence of secondary amenorrhea in athletes. One of the main factors is high energy expenditure associated with many athletic activities, which can lead to energy deficits and disrupt regular ovulatory cycles. In addition, many athletes engage in disordered eating practices such as restricting food intake or engaging in extreme dieting to maintain leanness for optimal performance (1). Another potential factor is psychological stress associated with athletic training and overstated goals.

Concerning stress-related FHA (3), the Endocrine Society Clinical Practice Guidelines (14) claim that various types of stress and life events, which are perceived as traumatic and/or stressful experiences by women, might trigger the disease. In this sense, it has been argued that FHA is a type of somatic disorder caused by stress (15–17). Russell and colleagues (18) identified a wide spectrum of stressors that may characterize the prior experience of women with FHA, such as extreme danger, fear for one’s own safety profound family conflict or loss, and minor life changes (starting school, etc.). So far, a fair number of literature reviews has been published investigating the pathophysiological factors of FHA or its consequences also in term of psychological effects (7, 19, 20), However, to the best of our knowledge, no review has been specifically designed to address psychological factors in FHA, nor their role on the onset, persistence, or subtype of FHA. The primary aim of this systematic review was to assess the associations of psychological factors with the presence (cases) vs the absence (controls) of FHA. The recognition of the involvement of such factors may help the identification of new clinical strategies for the management of FHA.

## 2 Materials and methods

The systematic review was conducted according to the Cochrane Collaboration guidelines (21) and the PRISMA Statement (22), to provide a comprehensive and unbiased overview of the evidence defining psychological factors associated with FHA, its onset or persistence.

### 2.1 Search strategy

To include the broadest range of literature, the electronic literature search was conducted on the four major databases in the field of health sciences: PubMed, PsycINFO, Scopus, and Embase.

**Figure 1:**
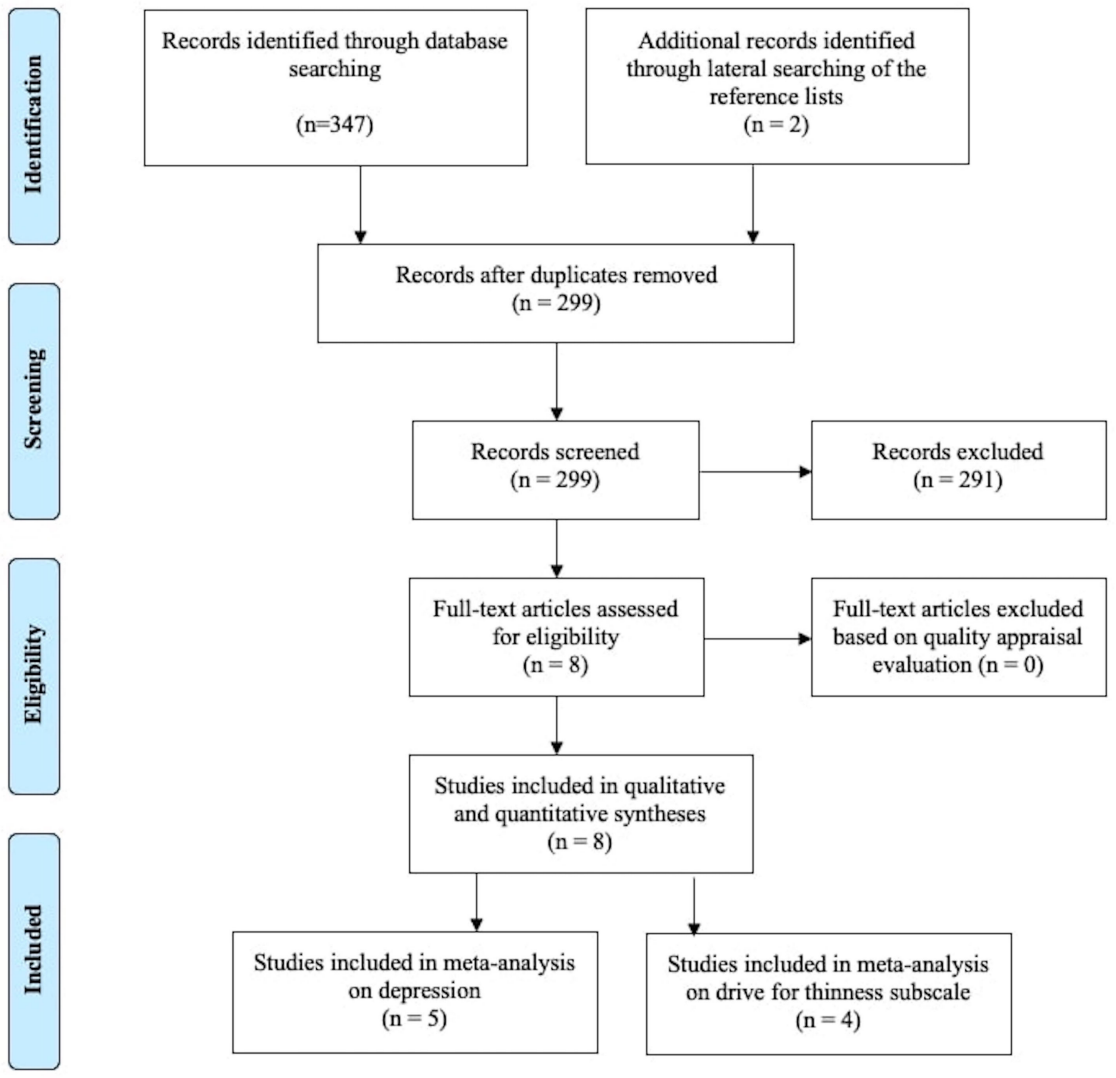
Flow diagram of literature search and process of studies’ selection.

The search was limited to articles published from 2000 onwards (in order to focus on more contemporary psychological constructs and assessments) and to English-language journal articles. The literature search was undertaken on the 24th of February 2022 and was adapted for each database as necessary. The search strategy includes Medical Subject Headings (MeSH) and text words for the following domains of interest: 1) secondary amenorrhea/secondary amenorrhoea/functional hypothalamic amenorrhea; AND 2) psychology/psychological/mental health, OR 3) stress/stressor/distress, OR 4) emotions/emotional/affective/alexithymia. The selection of the search term was based on the literature on FHA and the clinical experience. Lateral searching of Google Scholar and of the reference lists of included studies was executed manually. An expert librarian was consulted to verify that the search strategy was adequate to yield a comprehensive literature search. **Figure 1** shows the process of literature search and selection of publications.

### 2.2 Eligibility criteria

The inclusion criteria were as follows:

- diagnosis of FHA based on current criteria (1);
- peer reviewed studies;
- studies adopting an analytical study design (i.e., an observational study with a control or comparison group);
- studies addressing psychological dimensions using standardized and validated instruments.

Exclusion criteria were:

- intervention studies;
- non-empirical studies (e.g., case reports, commentaries, reviews, abstract meeting, or letters);
- studies not addressing psychological dimensions;
- not available full text.

### 2.3 Study selection and data extraction

Study selection was performed independently by two researchers (FB, LB), through the screening of records (i.e., titles, abstracts, and keywords). If the abstract was insufficient to determine the eligibility of the article, screening was based on the full text. When the two researchers did not reach a consensus on inclusion, decisions were made through discussion with a third researcher (GP).

A standardized data form was prepared in Excel to simplify data management. The following information was extracted for each eligible article: title, authors, country of the first author, keywords, article type.

### 2.4 Quality assessment

The “Quality appraisal checklist – quantitative studies reporting correlations and associations” by the National Institute for Health and Clinical Excellence (NICE) (23) was used to assess quality of studies. Two reviewers (FB, LB) independently applied criteria to assess a study’s internal and external validity and address the following key aspects of study design: characteristics of study participants; definition of independent variables; outcomes assessed, and methods of analyses. Checklist items are worded so the following responses are possible: “++” =2; “+” = 1; “-” = 0., “Not Reported (NR)” = 0. Since Items 3.4 and 3.5 are not applicable to any of the included studies, they were not considered in the total count. For each article, the sum of all scores was calculated; the highest possible score was 34. The threshold for high quality was set at 75% (minimum score=25.5) of the total score. Disagreements were resolved through discussion with a third researcher (GP).

The quality assessment did not eliminate any studies, rather it provided a comprehensive view of the quality of the studies’ methodology, analysis, and presentation of results (**Supplementary Table A**).

### 2.5 Meta-synthesis and meta-analysis

First, a meta-synthesis was undertaken to synthesize evidence and provide a comprehensive overview of the data. Thus, two reviewers (FB, LB) independently identified psychological dimensions based on psychological constructs (e.g., depression, anxiety, etc.) describing the findings that emerged from the included studies. Data were clustered and summarized to describe the identified dimensions.

The primary analysis was calculated using descriptive statistics reported in the results section of each study and involved differences in the distributions of psychological factors between FHA women and controls (or comparison). Since all studies compare mean values and standard deviations (SDs) across case/control groups on continuous outcome variables, effect sizes (expresses as mean difference) and the corresponding 95% confidence interval (CI) were performed using Cohen’s d (24) or Hedge’s g (25) and its 95% confidence interval (26) for each outcome measure. Specifically, Cohen’s d was calculated when two groups have similar standard deviations and are of the same size; while Hedges’ g was computed when there are different sample sizes. Cohen’s d or Hedge’s g values were interpreted as small if 0.2≤ d (or g) < 0.5, medium if 0.5 ≤ d (or g) < 0.8; large if d (or g) ≥ 0.8 (27).

Then, because the included studies were heterogeneous in terms of variables and instruments, it was not always appropriate to undertake a meta-analysis (21). A decision was made to only conduct a meta-analysis if four or more studies assessed the same psychological construct, as already made in previous studies (28).

Meta-analysis was performed using the Comprehensive Meta-Analysis Software program (26).

Statistical heterogeneity was assessed with the I^2^, which has been conventionally adopted to indicate low, moderate, and high heterogeneity values of 25%, 50% and 75%, respectively (29). As heterogeneity among studies was expected on the basis of large variability in the assessment of psychological constructs across different studies, both fixed and random-effects models were used (30).

To investigate potential publication bias, the funnel plot of the results of the included studies was checked for asymmetry (31).

## 3 Results

### 3.1 Study characteristics

Of the 349 publications found, 347 were identified by database search and 2 by an additional search based on the articles’ bibliography. The process of removing duplicates and screening titles and abstracts resulted in 8 publications. Therefore, we included 8 studies in the review (**Table 1**), of which two (36, 37) refers to the same study population (and were therefore included but jointly analyzed). All studies used a case-control design (women with FHA vs healthy controls), with 3 studies also included a comparison group (organic amenorrhea or due to anorexia nervosa -AN-), for a total of 514 women: 209 with FHA, 239 eumenorrheic controls, and 66 with other forms for amenorrhea.

**Table 1:** Characteristics of the studies included in the review.

Author (year); Country	N sample Mean age, SD	N control/comparison (Mean age, SD)	Variables observed	Measure	FHA versus controls [Effect size (CI 95%)]	FHA versus comparisons [Effect size (CI 95%)]
**Marcus et al.** (32)**; USA**	28 with FHA 26.4 ± 5.6	24 with organic amenorrhea 26.2 ± 4.7 25 eumenorrheic 25.9 ± 5.1	Depression	Beck Depression Inventory Hamilton Raing Scale for Depression	Higher levels of depressive symptoms both in Beck Depression Inventory [d= 0.77 (0.21-1.32)] and Hamilton Raing Scale for Depression [d= 0.74 (0.19 -1.30–)]	NS
			Disfunctional Attitude	The Disfunctional Attitude Scale	Higher dysfunctional attitudes [d= 0.83 (0.27-1.40)]. Specifically, greater need for approval [d=0.85 (0.29-1.41)]	NS
			Sefl-control	Self-Control Scale	NS	NS
			Eating attitude	Eating Disorder Inventory-2 Bulimia Test	Higher drive for thinness [d=1.17 (0.41-1.94)] Higher bulimia [d=1.12 (0.36-1.88)] Higher ineffectiveness [d = 0.77 (0.04-1.50)] Higher interoceptive awareness [d=0.90 (0.16-1.64)] Higher bulimic symptoms [d=1.42 (0.63-2.21)]	Higher drive for thinness [d =0.89 (0.20-1.59)] Higher bulimia [d=0.04 (-0.66-0.67)] Higher ineffectiveness [d=0.91 (0.21-1.61)] Higher interoceptive awareness [d==0.73 (0.05-1.42)] Higher bulimic symptoms[d==0.91 (0.22-1.61)]
**Bomba et al.** (11)**; Italy**	20 with FHA (10 N-FHA and 10 Hy-FHA) 16.4 ± 0.9	20 eumenorrheic 17.1 ± 1.2	Depression	Children’s Depression Inventory	NS	NA
			Eating attitudes	Eating Disorder Inventory-2;	N-FHA: Higher drive for thinness [g = 0.30 (-0.45-1.07)] Hy-FHA: Higher Ineffectiveness [g=0.67 (-0.11-1.45)] Higher Ascetism [g =0.71 (-0.07-1.49)] Higher Impulse regulation [g=0.26 (-0.50-1.02)] Higher Social Insecurity [g= 0.71 (-0.07-1.48)] Higher EDI-2 total score [g= 0.52 (-0.24-1.29)]	
			Previous trauma events and life events, psychosomatic disorders, and depressive and anxiety traits	Psychodinamic semistructured interview for girls and their parents	Mild subclinical depressive trait Subclinical eating disorders in adolescence Stressfull events were reported by parents of FHA patients; mainy, precence of psychoatric disease in famiy, postpartum depression, intrafamiliar conflicts, transferts, and chornic disease Reported life events associated with the onset of amenorrhea were common life events that these teenagers felt as highly stressful (such as change of school or breaking up with boyfriend). Eighty percent of FHA girls reported having an excessively demanding, controlling, and intrusive mother, with conflicting family relationships in 50% of cases.	
**Dundon et al.** (33)**; USA**	41 with FHA 26.1 ± 5.5	39 eumenorrheic 25.1 ± 4.9	Sexual function	McCoy Female Sexuality Questionnaire	Lower scores on sexuality [d = 1.22 (0.74-1.69)]	NA
			Depression	Zung Self-Rating Depression Scale	Higher depression [d = 0.83 (0.37-1.29)]	
			Anxiety	Zung Self-Rating Anxiety Scale	Higher anxiety [d =1.26 (0.79-1.75)]	
**Bomba et al.** (34)**; Italy**	21 with FHA 16.2 ± 0.9	21 with anorexia nervosa 15.9 ± 1.1 21 eumenorrheic 16.2 ± 1.1	Depression	Children’s Depression Inventory;	Higher scores of depression at CDI [d =1.25 (0.59-1.90)]	Lower scores at CDI [d =-0.99 (-1.63 - -0.35)].
			Eating attitude	Eating Disorder Inventory-2	Higher drive for thinness [d=1.21 (0.55 – 1.87)] Higher maturity fears [d=1.30 (0.63-1.96)] Higher social insecurity [d = 0.94 (0.30-1.58)]	Lower drive for thinness [d= -0.78 (-1.41–0.16)] Lower ineffectiveness [d= -0.65 (-1.27- -0.03)]; Lower interpersonal distrust [d=-0.87 (-1.50 - -0.24)] Lower interoceptive awareness [d=-1.08 (-1.73 - -0.44)]
			Alexithymia	Toronto Alexthymia Scale-20	Higher scores of alexithymia [d= 1.27 (0.61-1.93)]; and the difficulties in describing feelings subscale [d = 1.38 (0.71-2-05)]	Lower scores at difficulty in identifying feelings [d= -0.94 (-1.58- -0.30)]
**Pentz and Radoš** **(** 35)**; Croatia**	25 with FHA 21 (18-24)^1^	21 with organic amenorrhea 23 (21 - 26)^1^ 20 eumenorrheic 24 (20.5 – 26.5)^1^	Trait measurements	Multidimensional Perfectionism Scale Self-Control Scale	Higher levels of perfectionism trait [d= 1.39 (1.06-1.72)] Higher levels of concerns over mistake [d= 1.08 (0.85-1.31)]	Higher levels of personal standards [d= 0.73 (0.61-0.85)]
			Eating attitudes	Adolescent Dieting Scale + Eating Attitude Test	NS	NS
			Parental rearing perception	Memories of upbringing	NS	NS
			DSM-IV Disorders	Structured Clinical Interview for DSM-IV Disorders (SCID)	History of anorexia nervosa	History of anorexia nervosa
**Tranoulis et al.** **(** 36, 37)**; UK**	41 with FHA (17.8 ± 1.8)	86 eumenorrheic (18.3 ± 2.7)	Sleep disorders^a^	Athens Insomnia Scale^a^	Higher scores at the following subscales of AIS-8^a^ [g= 0.55 (0.17-0.93)]: awakenings during the night [g== 0.71 (0.32-1.09)] final awakening [g=1.13 (0.73-1.52)] total sleep duration [g= 0.65 (0.27-1.03)] quality of sleep [g= 1.6 (1.18-2.02)]. Lower sleepiness during the day^a^ [g=-1.0 (-1.39 - -0.61)]	NA
			Anxiety^a,b^	State-Trait-Anxiety-Inventory^a,b^	Higher scores of STAI g=1.10 (0.70 - 1.50)]	
			Eating Attitudes^a,b^	Eating Attitude Test-26^a,b^	Higher scores of EAT-26 [g= 1.36 (0.95-1.77)]^a^ Higher scores of EAT-26 subscales^b^: specifically dieting [g= 0.90 (0.51-1.29)], bulimia and food preoccupation [g= 1.72 (1.29-2.15)].	
			Overweight preoccupation^a,b^	Multidimensional Body-Self-Relations Questionnaire^a,b^	Higher scores of MBSRQ [g= 1.06 (0.67-1.45)],	
			Physical Activity^a,b^	International Physical Activity Questionnaire^a,b^	Higher scores of IPAQ [g= 0.57 (0.19-0.94)].	
**Strock et al.** **(** 38)**; USA**	33 with FHA (21.2 ± 0.5)	28 eumenorrheic (24.1 ± 0.9)	Mood	Profile of Mood States	NS	NA
			Depression	Beck Depression Inventory	NS	
			Stress	Daily Stress Inventory Perceived Stress Scale	NS	
			Eating Attidues	Three-Factor Eating Questionnaire Eating Disorder Inventory	Higher drive for thinness [d= 1.00 (0.47-1.54)] Higher cognitive restraint [d= -4.16 (3.26 -5.05)]	
			Disfunctional Attitude	Dysfunctional Attitude Scale	Greater need for social approval [d= 2.7 (2.00 – 3.39)]	
			Resilience	Brief Resilient Coping	NS	

^1^Data refers to median and interquartile range.

*The appropriate Effect size measure for each variable is calculated. Specifically, Cohen’s d is reported when two groups have similar standard deviations and are of the same size. While Hedges’ g is calculated when there are different sample sizes.

### 3.2 Psychological factors

The psychological variables detected by the selected studies are described in **Table 2**.

**Table 2:** Psychological assessment.

Topics of assessment	Questionnaire	Notes for use and studies specificities
***Alexithymia***	Toronto Alexithymia Scale-20 (TAS-20) (39)	Toronto Alexithymia Scale-20 (TAS-20) is a 20-item self-reported questionnaire which evaluates the presence of alexithymia. It uses a 5-point Likert scale. TAS-20 measures 3 dimensions: difficulty in identifying feelings, difficulty in describing feelings to others, and externally oriented thinking. The presence of alexithymia is defined when the score is over 61; scores from 51 to 60 are considered as at risk (39).
***Anxiety***	*State-trait Anxiety Inventory (STAI)* (40)	The STAI is a 20-item self-report questionnaire which assesses the levels of state and trait anxiety. It uses a 4-point Likert scale from “not at all” to “very much”. The STAI consists of two separate self-report scales each containing 20 questions. The first one evaluates how the patients ‘currently feel’ (state anxiety). The second one assesses how they ‘generally feel’ (trait anxiety). The total score on both subscales ranges from 20 to 80, with higher scores indicating greater levels of anxiety. Cut-off points is 40 on either subscale for all the translations (40).
	*Zung Self-Rating Anxiety Scale* (41)	The Zung Self-Rating Anxiety Scale is a 20-items self-report inventory which evaluates the anxiety severity. Each of the 20 items is scored using a 4-point scale ranging from 1 to 4. The anxiety score ranges from 20 to 80. In clinical screening, the recommended cut-off is the index scores of 36. In research, the cut-off of 40 would be most appropriate. (41, 42)
***Axis I mental disorders***	*SCID-I-RV* (43)	SCID-I-RV is a structured clinical interview created for making DSM-IV Axis I mental disorders diagnosis. It resumes the modules related to the DSM-IV sections.
***Depression***	*Zung Self-Rating Depression Scale* (44)	The Zung Self-Rating Depression Scale is a 20-items self-administered survey which assesses depression symptoms. The depression score ranges from 25 to 100. For recognizing adults with depressive disorder, the recommended cut-off is index scores of 50 and over (44).
	*Children’s Depression Inventory (CDI)* (45)	Children’s Depression Inventory (CDI) is a 27 items self-report questionnaire which assesses symptoms of depression in children and adolescents. Total score ranges from 0 to 54. The scores of 19 are considered the cut-off beyond which the subject is considered characterized by depressive symptoms, while the scores of 17 and 18 detect subjects at risk (45).
	*Beck Depression Inventory* (46)	The Beck Depression Inventory is a 16-item self-report questionnaire that evaluates the severity of depression. Scores ≥ 10 are representative of clinically significant depressive symptoms (46).
	*Beck Depression Inventory-II* (47)	Beck Depression Inventory-II is a 21-item self-report questionnaire which measures the severity of depressive symptoms. The questionnaire follows the criteria for major depressive disorder following the fourth edition of the Diagnostic Statistical Manual. Cut-off scores are: scores of 14–19 for mild depression, scores of 20–28 for moderate depression, scores ≥28 for severe depression (47).
	*Hamilton Rating Scale for Depression* (48)	The Hamilton Rating Scale for Depression is a 17-items clinical rating scale which assesses the severity of depression. The test is based on a Likert scale of either 0 to 4 or 0 to 2. Scores can range from 0 to 54. For identifying depression, scores ≥ 14 are used as a reference (48).
	*Dysfunctional Attitude Scale* (49)	The Dysfunctional Attitude Scale is a 40-item self-report scale that assesses the presence of dysfunctional attitudes usually held by persons predisposed to depression. Each item consists of a 7-point Likert scale (7 = fully agree; 1 = fully disagree). The score is the sum of the 40-items with a range from 40 to 280; higher scores are representative of greater dysfunction (49).
***Eating attitudes and disorders***	*Bulimia Test—Revised (BULIT-R)* (50)	The Bulimia Test-Revised is a 36-item self-report questionnaire which evaluates symptoms of bulimia nervosa and binge eating. Items are presented in a 5-point Likert scale. Scores are obtained by summing responses. Scores ≥104 can be used as a cut-off to indicate diagnosable bulimia nervosa (50).
	*Eating Attitude Test (EAT-26)* (51).	The EAT is a 26-items self-report questionnaire which assesses symptoms of eating disorders. It includes three sub-scales: dieting (13 items), bulimia and food preoccupation (6 items), oral control (7 items). It uses a six-point scale from “never” to “always”. The score ranges between 0 and 78. The presence of abnormal eating behavior is defined when the score is at or above 20 (51).
	*Multidimensional Body-Self Relations Questionnaire (MBSRQ)* (52)	The Multidimensional Body-Self-Relation Questionnaire is a 69-item self-report inventory which assesses self-attitudinal aspects of the body-image. Possible answers are organized on a five-point scale (from “definitely disagree” to “definitely agree”). A total score at or above 2.5 confirms the presence of Overweight Preoccupation. The MBSRQ was translated and validated in Greek population (52).
	*Three Eating Factor Questionnaire* (53).	The TFEQ is a 51-item self-administered questionnaire which evaluates three dimensions of eating behavior. The TFEQ consists of three subscales: cognitive restraint (0-10 low, 11-13 high, 14-21 clinical range) disinhibition, and perceived hunger. The answers are scored 0 or 1 and must be added together (53)..
	*Eating Disorder Inventory-2 (EDI-2)* (54)	Eating Disorder Inventory-2 is a 91 item self-report questionnaire which assesses eating attitudes usually associated with eating disorders. The 91 items are on a 6-point Likert scale, from never to always, and are divided into 11 main subscales: drive for thinness (DT), bulimia (BU), body dissatisfaction (BD), ineffectiveness (IN), perfectionism (P), interpersonal distrust (ID), interoceptive awareness (IA), maturity fear((MF), asceticism (ASC), impulse regulation (IR), social insecurity (SI). Subscale scores are obtained by adding all item scores on each subscale Each item can be score with a scoring system. It transforms scores ranging from 0 to 3 rather than 0 to 5: a score from 1 to 3 is considered as a “symptomatic” response (always= 3, usually=2, and often = 1), and 0 is assigned to the three “asymptomatic” responses (sometimes, rarely and never (54).
	*The Adolescent Dieting Scale (ADS)* (55)	The Adolescent Dieting Scale is a 8 item questionnaire that assesses three strategies of dieting: calorie counting, decrease of food intake and skipping meals. It uses a four-point scale from 0 (never) to 3 (almost always). The total score ranges from 0 to 24. A score from 1 to 6 indicates the presence of minimal dieting, a score from 7 to 14 indicates intermediate dieting, and a score of 15 or higher indicates extreme dieting (55).
***Mood states***	*Profile of Mood States (POMS)* (56)	The Profile of Mood States (POMS) is a 65-item self-report questionnaire which evaluates transient mood states. The POMS examines six different mood dimensions: anger-hostility, confusion-bewilderment, depression-dejection, fatigue-inertia, tension-anxiety, and vigour-activity. The presence of a good mood is confirmed by high scores in the vigour subscale and low scores in the five other subscales. A Total score is calculated by summing the totals for the negative subscales (tension, depression, fatigue, confusion, anger) and subtracting the totals for the positive subscales (vigor and esteem-related affect) (56).
***Perfectionism***	*Multidimensional Perfectionism Scale* (57)	The Multidimensional Perfectionism Scale is a 35-item self-report questionnaire that assesses different levels of multidimensional perfectionism. All the items are organized in six subscales: Concern over Mistakes (9 items), Personal Standards (7 items), Parental Expectations (5 items), Parental Criticism (4 items), Doubts about Actions (4 items) and Organisation (6 items). Each item is rated on a five-point Likert Scale from 1 (disagree completely) to 5 (agree completely). The total score ranges from 35 to 175: a higher score is representative of higher levels of perfectionism (57).
***Physical activity***	*International Physical Activity Questionnaire (IPAQ)* (58)	The International Physical Activity Questionnaire is a 27-items self-report questionnaire that measures the physical activity in adult patients aged 15 to 69 years old. There are 3 possible levels of physical activity: low, moderate, or high. The questionnaire was translated and validated in a Greek population (58, 59)..
***Self-control***	*Self-control Scale* (60)	The Self-Control Scale is a 36-item measure which assesses the ability to cope or learned resourcefullness. It uses a 5-point scale. Higher values indicate greater self-control (60).
***Sexual function***	*Italian McCoy Female Sexuality Questionnaire* (61)	MFSQ-I is a questionnaire which assesses sexual function. It is divided into two factors supported by principal component analysis: Sexuality, called MFSQ-Sex (9 items) and Partnership, called MFSQ-Partner (5 items). The sexuality factor (range of scores 9–49), includes items on sexual desire, sexual arousal, orgasm, and satisfaction or enjoyment of sexual activities. The partnership factor (range of scores 5–35), includes items about satisfaction with partner as a lover, emotional closeness achieved with partner, and feeling attractive to one’s partner. In both factors, higher scores indicate higher levels of function (61).
***Resilience***	*Brief Resilience Scale* (62).	Brief Resilience Scale is a 6-item self-rating questionnaire that assesses the ability to recover from stress. It examinates personal characteristics like resilience, coping styles, social relationships, and health-related outcomes. The total score is obtained adding the value (1–5) of responses, creating a range from 6-30 and dividing the sum by the total number of questions answered. The presence of low resilience is defined when the score is under “3” (62).
***Stress***	*Daily Stress Inventory* (63)	Daily Stress Inventory (DSI) is a 58-item self-report measure that assesses the stressfulness of events occurred during the last 24-hour period. It generates three daily scores: a frequency score, an impact score, an average impact rating. They are calculated by dividing the sum of impact rating by the frequency of events (63).
	*Perceived Stress Scale* (64)	Perceived Stress Scale is a 14- item self-report questionnaire which assesses individual stress levels. It uses a five-point Likert scale, from “never” to “very often”. Total score ranges from 0 to 56. Scores are obtained by reversing the scores on the seven positive items and then summing. (64).
***Perceptions of parental rearing behaviors***	*Egna Minnen av Barndoms Uppfostran; one’s memories of upbringing (EMBU))* (65)	Memories of upbringing is 81 item self-report questionnaire evaluating the own memories of parental rearing behavior. Each item is rated on a four-point Likert scale from 1 (never) to 4 (always). The study of Pentz and Radoš adopted a shorter version of 23 items that is considered as a valid equivalent cross-culturally instrument. It consists of three subscales: parental Rejection (7 items), Emotional Warmth (6 items) and Overprotection (9 items). Two forms are given, one for perception of mother’s behaviors and one of father’s behaviors (65)..
***Sleep***	*Athens Insomnia Scale* (66)	The Athens Insomnia Scale is an eight-item self-report questionnaire which assesses sleep difficulty. It is rated on a 0-3 scale. A cut-off of 6 indicates the diagnosis of insomnia. The questionnaire is validated in a Greek adult population. (66)

The psychological aspects assessed are numerous, including both symptomatologic dimensions as depression, anxiety, stress, sleep disorders, or eating disorders, and more stable or trait dimensions as alexithymia, self-control, perfectionism, need for social approval, or resilience. Sexual functioning is included in one study.

### 3.3 Depression

Five studies (11, 32–34, 38) evaluated the role of depression in FHA using self- report instruments.

We observed heterogeneity in the populations involved and in the questionnaires adopted in each study. Three studies involved adult women (32, 33, 38) and assessed depression through the Beck Depression Inventory (32, 38) or the Zung Self-Rating Depression Scale (33); while two studies involved FHA adolescents assessing depression through the Children Depression Inventory (11, 34).

Considering depression, a significant difference in level of depression between FHA patients and controls was found both in adult (32, 33) and adolescent (34) populations (respectively: d = 0.77 [0.21 − 1.33]; d = 0.83[0.37 − 1.29]; d = 1.25 [0.59 − 1.90]). In contrast to these results, the study by Pentz and colleagues (38) did not find a significant difference between FHA women and healthy controls, neither on the scale of depression (BDI) nor with the scale of mood disorders (Profile of Mood States).

**Figure 2:**
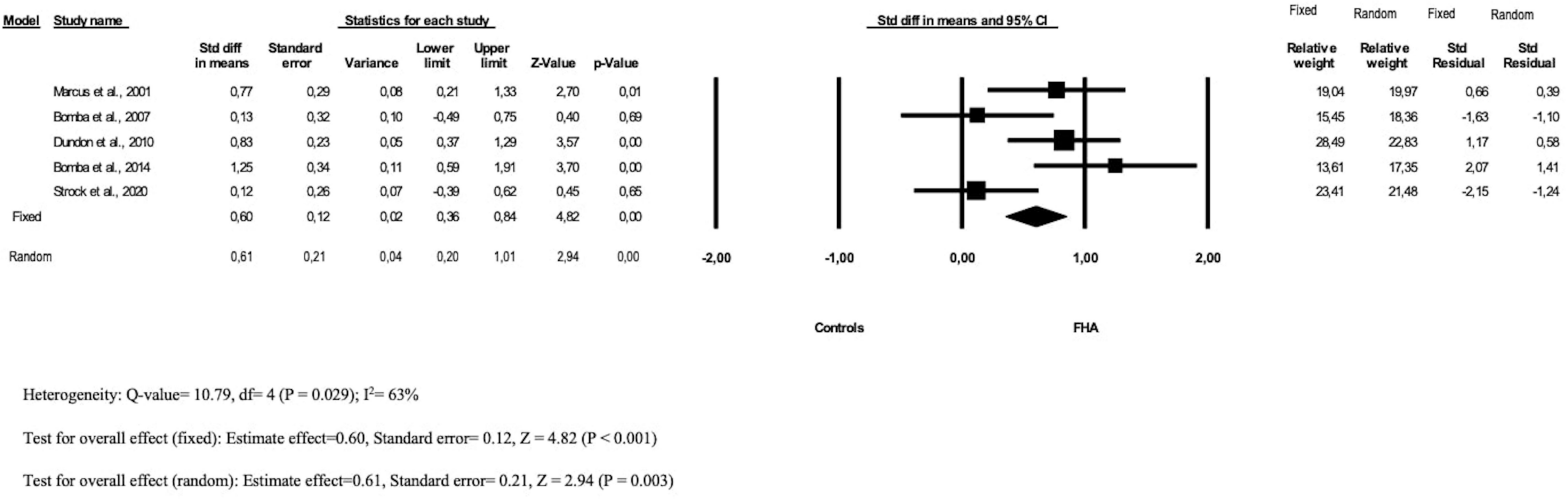
Forest plot of depression prevalence in FHA patients and controls.

The pooled MD across the studies was statistically significant both in the fixed 0.60 (95% CI: 0.36–0.84) and random model 0.61 (95% CI: 0.20–1.01); the heterogeneity between the studies included was moderate (I^2^ 63%) (**Figure 2**).

The funnel plot (**Supplementary Figure A**) showed a low risk for publication bias.

Considering the comparison between FHA patients and women with other amenorrhoeic conditions, a non-significant difference was found between FHA patients and patients with organic amenorrhea (32) while a significant difference was found between FHA patients and AN patients (d = -0.99[-1.63 − -0.35]) (34), with FHA adolescents reporting less depressive symptoms when compared to adolescents with AN.

### 3.4 Eating disorders

Seven studies (11, 32, 34–38) evaluated the role of eating attitudes and eating disorders in FHA using self- report instruments. Specifically, four studies used the Eating Disorder Inventory-2 (EDI-2) (11, 32, 34, 38) finding differences between FHA patients and controls or comparison groups with respect to the EDI-2 subscales (see **Table 2** for all data). In particular, the subscale drive for thinness, which assesses excessive concern with dieting, preoccupation with weight, and fear of weight gain, was found to be significantly higher in FHA patients when compared to controls or organic amenorrhoeic patients, with large effect sizes (32, 34, 38), and significantly lower when compared to patients with AN, with moderate effect sizes (34).

**Figure 3:**
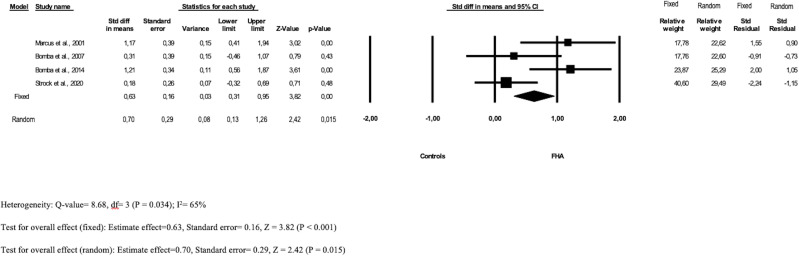
Forest plot of drive for thinness subscale prevalence in FHA patients and controls.

The meta-analysis on the subscale drive for thinness (**Figure 3**) revealed that the pooled MD across the studies was statistically significant both in the fixed 0.63 (95% CI: 0.31–0.95) and random model 0.70 (95% CI: 0.13–1.26); the heterogeneity between the studies included was moderate (I2 65%). The funnel plot (**Supplementary Figure B**) showed a low risk for publication bias.

Moreover, two studies (35–37) used the Eating Attitude Test-26, with contrary findings: while Tranoulis and colleagues (36, 37) found a significant difference in EAT-26 scores between FHA patients and controls (g= 1.36 [0.95-1.77]), and specifically higher dieting [g= 0.90 (0.51-1.29)], bulimia and food preoccupation [g= 1.72 (1.29-2.15)], the study by Pentz & Rados (35) did not found difference in disordered eating behaviors between FHA and controls; however, FHA patients reported more prevalent history of anorexia nervosa (20% vs 0% and 0%, respectively).

### 3.5 Other psychological symptomatologic dimensions: anxiety, stress, sleep disorders, psychiatric disorders

Two studies assessed anxiety, both in term of level of state (current) and trait (lifetime) (33, 36, 37), using two different measurement scales (Zung Self-rating Anxiety Scale and State-Trait Anxiety Scale respectively). Moreover, the study of Strock and colleagues (38) explore anxiety symptoms through a subscale of the Profile of Mood States.

A significant difference in level of anxiety between FHA patients and controls was found (33, 36, 37) (respectively: d = 1.26 [0.79 − 1.75]; g = 1.10 [0.70 − 1.50]). Higher prevalence was shown among FHA patients.

The study by Strock and colleagues (38) assessed the role of stress through two self-reported questionnaires, without showing significant difference between FHA patients and controls.

One study (36) explored the presence of sleep disorders finding significant differences between FHA patients and controls regarding sleep problems (see **Table 2**).

Two studies explored psychopathological dimensions by clinical interviews, one using a psychodynamic semistructured interview (11) and one using the Structured Clinical Interview for DSM-IV Disorders (SCID) (35), with miscellaneous results (see **Table 2** for all details). Briefly, Bomba and colleagues (11) showed that FHA patients reported: i) an absence of important depressive and anxious disorders in FHA patients, similarly to control subjects; ii) a presence of mild subclinical depressive traits in FHA patients; iii) a presence of subclinical eating disorders in adolescence; iv) presence of previous stressful events reported by their parents and presence of common life events associated with the onset of FHA.

Also, the study by Pentz & Rados (35) revealed that the prevalence of depressive or anxiety disorders did not differ between FHA and control or comparison groups, while FHA patients reported more prevalent history of anorexia nervosa (20% vs 0% and 0%, respectively).

### 3.6 Psychological trait dimensions: alexithymia, dysfunctional attitudes, parental rearing perception

The study by Bomba and colleagues (34) assessed alexithymia and found a significant difference between FHA adolescents and healthy controls in relation to alexithymia and one of its subscale reflecting difficulties in describing feelings [respectively d= 1.27 (0.61-1.93) and d = 1.38 (0.71-2-05)]. FHA adolescents reported higher levels of alexithymia.

Three studies assessed other dysfunctional attitudes as self-control, perfectionism, and need for social approval (32, 35, 38), finding that FHA women reported higher dysfunctional attitudes [d= 0.83 (0.27-1.40)] (32) and, specifically: a significant effects on a greater need for approval (32, 38) [respectively, d=0.85 (0.29-1.41) and d= 2.7 (2.00 – 3.39)], higher levels of perfectionism trait [d= 1.39 (1.06-1.72)] and higher levels of concerns over mistake [d= 1.08 (0.85-1.31)] compared to controls (35). No difference was found regarding self-control neither compared to control (32, 38, 43) nor to comparison (32, 35) groups.

Two studies assessed the parental rearing perception, one with a questionnaire (35) and one through a clinical interview (11) with contrasting findings. While the study by Pentz & Rados (35) showed that the FHA group did not differ from controls in perception of parental rejection, emotional warmth or overprotection, the study by Bomba and colleagues (11) found that 80% of FHA girls reported having an excessively demanding, controlling, and intrusive mother, with conflicting family relationships in 50% of cases.

### 3.7 Sexual functioning

The study by Dundon and colleagues (33) also explore the sexual functioning of FHA patients, showing a significant difference on sexuality between FHA patients and controls [d = 1.22 (0.74-1.69)], but not on satisfaction with partner. Specifically, FHA women reported lower scores on sexuality scale that includes sexual desire, sexual arousal, orgasm, and satisfaction or enjoyment of sexual activities when compared to controls

## 4 Discussion

FHA is a form of secondary amenorrhea with a complex etiology, in which numerous factors have been implicated as specific triggers for its onset. In this review, we present an overview of the psychological factors associated with FHA.

All reviewed studies found that psychological factors played a role in FHA condition. Despite the heterogeneity of studies and populations, we identified some recursive psychological factors that might be clinically relevant. It is possible to divide findings in two main categories: those exploring psychological state, which might be transitory, like depression, eating attitudes, anxiety, and sleep disturbances; those focusing on psychological traits, which may represent benchmark index of patients functioning, like alexithymia, stress, and life events.

As for the symptomatologic dimensions, studies found numerous significant results on psychological state of FHA patients, identifying depression and eating attitudes as the most common psychological disturbances in FHA patients. A significant level of mood symptoms and depressive scores was showed among FHA women and adolescents (11, 34). Women with FHA experienced significantly higher depression than healthy women (33). However, two studies (11, 35) assessing psychopathological dimensions by clinical interviews, showed that FHA patients do not manifest important depressive disorders, with scores similar to those of control subjects. According to these findings, patients with FHA may often manifest depressive symptoms; however, they do not satisfy criteria for a clinical diagnosis for depressive disorder (11, 35).

Eating attitudes and behaviors were investigated, finding difference between FHA patients and controls/comparison groups. These findings are in line with previous studies (13, 67) showing that several dysfunctional attitudes towards nutrition, such as a focus on diet, extreme physical activity, and fear of gaining weight, characterize the psychological profile of FHA patients. Dysfunctional eating attitudes and subthresholding restrictive anorexia nervosa seem to be associated with FHA (11). Although patients with FHA do not meet the psychopathological criteria for a diagnosis of AN, they reported a higher prevalence of previous diagnosis of anorexia nervosa (35).

Overall, it is noteworthy that the symptoms that emerged *via* self-report were not confirmed by the interviews performed (11, 35). Further studies are needed to assess psychological variables with instruments other than the self-report measures, which work well as screening measures but have no diagnostic purpose.

Furthermore, reviewed studies have investigated other psychopathological symptomatology. Studies by Dundon and colleagues (33) and Tranoulis and colleagues (37) found a significant difference in anxiety levels between FHA patients and controls, while the study by Strock et al. (38) did not identify any significant findings. One study (36) explored the presence of sleep disturbances, finding significant differences with a higher prevalence among FHA patients.

One of the most popular hypotheses on the etiology of amenorrhea is that stress is one of the important pathogenetic factors in FHA patients. Although this hypothesis has been confirmed over time (6, 15–17), surprisingly only one study has investigated the role of stress in the diagnosis of FHA, revealing no significant findings. This finding is in contrast with previous literature. For example, Berga and colleagues (68) in a case-control study found that the group consisting of women with FHA had a higher concentration of cortisol than eumenorrheic women due to stress. According to the authors, high cortisol levels characterize the clinical condition of patients with FHA and, considering the recognized association between cortisol and stress, they count stress among the factors involved in the onset of FHA (68). Specifically, a key endocrinological aspect of stress is hyperactivation of the hypothalamic-pituitary-adrenal axis (6). Stress increases the secretion of corticotropin-releasing hormone and consequently contributes to the elevated levels of adrenocorticotropin and cortisol secretion, which reduce GnRH drive (7). However, it is difficult to find a clear definition of what ‘stress’ means in relation to FHA. Lazarus and Folkman (69) claim that stress originates from those situations in which the individual perceives his/her own resources to be insufficient to cope with the situation. To date, the definition and the nature of the stress underpinning FHA are still an intriguing matter of debate. Thus, further studies should define stress related to FHA and clarify its role.

Concerning psychological traits, reviewed studies explored alexithymia, dysfunctional attitudes, and parental rearing perception, which can represent benchmark index of patients functioning. An interesting study (34), compared alexithymia levels between FHA adolescents and control, showing that FHA patients have more difficulties in describing feelings. Alexithymia refers to the difficulties that individuals gave in perceiving, differentiating, and expressing emotions. This condition often characterizes the psychological profile of patients with somatic disorders (70) and it could be also a characteristic of women with FHA. In the reviewed studies FHA women reported higher dysfunctional attitudes such as higher level of perfectionism, higher concerns over mistakes, and greater need for approval than controls (32, 35). This is in line with previous literature (13, 14, 68), which have defined the psychological profile of women with menstrual problems, identifying low self-esteem, introversion, fear of judgement, high expectations of themselves in addition to those identified by this review.

As for parental rearing perception, studies found contrasting results (11, 35). However, in one of two studies 80% of FHA girls reported an excessive, demanding, controlling and intrusive mother, with conflict family in 50% of cases. Furthermore, stressful life events, such as presence of psychiatric disease in family, post-partum depression, intrafamiliar conflicts, transfers, and chronic disease are often reported in patients with FHA and felt very stressful, having an impact on the FHA condition (18).

Still concerning relational variables, Dundon and colleagues (33) investigated satisfaction with partner relationships and sexuality. No differences emerged in partner satisfaction, but a study (33) showed a significant difference in sexuality between FHA patients and controls. FHA women reported lower scores on the sexuality scale including sexual desire, sexual arousal, orgasm, and satisfaction or pleasure with sexual activities than controls. Although sexuality appears to be a central aspect of the psychological functioning of women with FHA, only one study has explored it (33).

Findings showed the association of psychological factors with the diagnosis of FHA and recognized their involvement in the persistence of the disorder. A multidisciplinary approach is fundamental in the clinical management of FHA and should involve a collaborative process between gynecologists, clinical psychologists, and psychiatrists, from diagnosis to treatment. The condition of FHA requires attention because it may reflect psychological or psychiatric difficulties and/or have a serious impact on daily life and quality of life. The primary intervention for women with amenorrhea is often solely focused on physiological aspects, but evidence has shown the positive impact of psychological interventions in terms of disease outcomes. Berga and colleagues (71) showed that a cognitive behavioral intervention designed to minimize problematic attitudes was likely to result in resumption of ovarian activity than observation. Based on the literature and results of this review, it is noteworthy that a tailored behavioral psychological intervention offers an effective treatment option that complements the clinical approach aimed at the recovery of ovarian activity.

The review presents some limitations. The review is based on only 8 studies; the small number of studies testifies to the fact that there is still a paucity of data regarding the psychological factors associated with FHA. Likewise, the number of patients composing the samples involved in the included studies is small. The heterogeneity of the studies’ designs and instruments allowed the meta-analysis to be carried out on a limited number of variables. Moreover, different instruments have been used to measuring the same construct. Since no study presented a prospective design from the time of diagnosis onwards, it was not possible to identify causal relationships between psychological factors and FHA and draw clear conclusions on the role of psychological factors in FHA.

Based on these considerations, further studies, possibly including epidemiological studies with prospective designs, are needed to clarify the temporal patterns and causal relationships between psychological factors and FHA. Likewise, additional studies on FHA are warranted to explore the role of psychological factors other than eating attitudes and depression. Specifically, the nature and the role of the stress underpinning FHA should be explored.

## Author contributions

FB, GP, DL, EV, and LB were involved in the study conceptualization and methodology development. FB and GP performed the search. LB and FB conducted data analysis. FB, GP, and LB were involved in writing the original draft of the manuscript. EV and DL reviewed and edited the manuscript. All authors contributed to the article and approved the submitted version.

## References

[1] Gordon CM. Clinical practice. functional hypothalamic amenorrhea. N Engl J Med (2010) 363(4):365–71. doi: 10.1056/NEJMcp0912024 20660404

[2] Warren MP Fried JL. Hypothalamic amenorrhea. the effects of environmental stresses on the reproductive system: A central effect of the central nervous system. Endocrinol Metab Clin North Am (2001) 30(3):611–29. doi: 10.1016/s0889-8529(05)70204-8 11571933

[3] Meczekalski B Podfigurna-Stopa A Warenik-Szymankiewicz A Genazzani AR. Functional hypothalamic amenorrhea: Current view on neuroendocrine aberrations. Gynecol Endocrinol (2008) 24(1):4–11. doi: 10.1080/09513590701807381 18224538

[4] Biller BM Federoff HJ Koenig JI Klibanski A. Abnormal cortisol secretion and responses to corticotropin-releasing hormone in women with hypothalamic amenorrhea. J Clin Endocrinol Metab (1990) 70(2):311–7. doi: 10.1210/jcem-70-2-311 2153693

[5] Deligeoroglou E Athanasopoulos N Tsimaris P Dimopoulos KD Vrachnis N Creatsas G. Evaluation and management of adolescent amenorrhea. Ann N Y Acad Sci (2010) 1205:23–32. doi: 10.1111/j.1749-6632.2010.05669.x 20840249

[6] Liu JH Bill AH. Stress-associated or functional hypothalamic amenorrhea in the adolescent. Ann N Y Acad Sci (2008) 1135:179–84. doi: 10.1196/annals.1429.027 18574223

[7] Meczekalski B Katulski K Czyzyk A Podfigurna-Stopa A Maciejewska-Jeske M. Functional hypothalamic amenorrhea and its influence on women's health. J Endocrinol Invest. (2014) 37(11):1049–56. doi: 10.1007/s40618-014-0169-3 PMC4207953 25201001

[8] Reifenstein EC psychogenic or hypothalamic amenorrhea. Med Clin North Am (1946) 30:1103–14. doi: 10.1016/s0025-7125(16)35908-9 21001722

[9] Harlow BL Signorello LB. Factors associated with early menopause. Maturitas (2000) 35(1):3–9. doi: 10.1016/s0378-5122(00)00092-x 10802394

[10] Nappi RE Facchinetti F. Psychoneuroendocrine correlates of secondary amenorrhea. Arch Womens Ment Health (2003) 6(2):83–9. doi: 10.1007/s00737-002-0152-4 12720058

[11] Bomba M Gambera A Bonini L Peroni M Neri F Scagliola P. Endocrine profiles and neuropsychologic correlates of functional hypothalamic amenorrhea in adolescents. Fertil Steril (2007) 87(4):876–85. doi: 10.1016/j.fertnstert.2006.09.011 17274991

[12] Stewart DE. Reproductive functions in eating disorders. Ann Med (1992) 24(4):287–91. doi: 10.3109/07853899209149956 1389091

[13] Ryterska K Kordek A Załęska P. Has menstruation disappeared? functional hypothalamic amenorrhea-what is this story about? Nutrients (2021) 13(8):1–15. doi: 10.3390/nu13082827 PMC8401547 34444987

[14] Gordon CM Ackerman KE Berga SL Kaplan JR Mastorakos G Misra M. Functional hypothalamic amenorrhea: An endocrine society clinical practice guideline. J Clin Endocrinol Metab (2017) 102(5):1413–39. doi: 10.1210/jc.2017-00131 28368518

[15] Brown E Bain J Lerner P Shaul D. Psychological, hormonal, and weight disturbances in functional amenorrhea. Can J Psychiatry (1983) 28:624–8. doi: 10.1177/070674378302800806 6420035

[16] Gallinelli A Matteo ML Volpe A Facchinetti F. Autonomic and neuroendocrine responses to stress in patients with functional hypothalamic secondary amenorrhea. Fertil Steril (2000) 73(4):812–6. doi: 10.1016/s0015-0282(99)00601-9 10731545

[17] Chrousos GP. Stress and disorders of the stress system. Nat Rev Endocrinol (2009) 5(7):374–81. doi: 10.1038/nrendo.2009.106 19488073

[18] Russell GF. Premenstrual tension and "psychogenic" amenorrhoea: psycho-physical interactions. J Psychosom Res (1972) 16(4):279–87. doi: 10.1016/0022-3999(72)90011-6 4562858

[19] Podfigurna A Meczekalski B. Functional hypothalamic amenorrhea: A stress-based disease. Endocrines (2021) 2(3):203–11. doi: 10.3390/endocrines2030020

[20] Roberts RE Farahani L Webber L Jayasena C. Current understanding of hypothalamic amenorrhoea. Ther Adv Endocrinol Metab (2020) 11:1–12. doi: 10.1177/2042018820945854 PMC7418467 32843957

[21] Higgins JPT Green S. Cochrane handbook for systematic reviews of interventions. Version 5.1.0. Cochrane Collaboration (2011).

[22] Page MJ McKenzie JE Bossuyt PM Boutron I Hoffmann TC Mulrow CD. The PRISMA 2020 statement: an updated guideline for reporting systematic reviews. BMJ (2021) 372:1–9. doi: 10.1136/bmj.n71 PMC8005924 33782057

[23] National Institute for Health and Care Excellence. Methods for the development of NICE public health guidance. London: National Institute for Health and Care Excellence (NICE (2012). 27905711

[24] Cohen S. Psychosocial models of the role of social support in the etiology of physical disease. Health Psychol (1988) 7(3):269–97. doi: 10.1037//0278-6133.7.3.269 3289916

[25] Lenhard W Lenhard A. Computation of effect sizes. (2016). doi: 10.13140/RG.2.2.17823.92329

[26] Borenstein M Hedges L Higgins J Rothstein H. Comprehensive meta-analysis version 2. Englewood NJ: Biostat (2005).

[27] Rosenthal R Cooper H Hedges L. Parametric measures of effect size. In: Cooper H Hedges L, editors. The handbook of research synthesis. New York: Sage Foundation (1994). p. p.231–244.

[28] Linardon J Tylka TL Fuller-Tyszkiewicz M. Intuitive eating and its psychological correlates: A meta-analysis. Int J Eat Disord (2021) 54(7):1073–98. doi: 10.1002/eat.23509 33786858

[29] Higgins JP Thompson SG Deeks JJ Altman DG. Measuring inconsistency in meta-analyses. BMJ (2003) 327:557–60. doi: 10.1136/bmj.327.7414.557 PMC192859 12958120

[30] Greenland S Robins JM. Estimation of a common effect parameter from sparse follow-up data. Biometrics (1985) 41:55–68. doi: 10.2307/2530643 4005387

[31] Sterne JA Egger M. Funnel plots for detecting bias in meta-analysis: Guidelines on choice of axis. J Clin Epidemiol (2001) 54:1046–55. doi: 10.1016/S0895-4356(01)00377-8 11576817

[32] Marcus MD Loucks TL Berga SL. Psychological correlates of functional hypothalamic amenorrhea. Fertil Steril. (2001) 76(2):310–6. doi: 10.1016/s0015-0282(01)01921-5 11476778

[33] Dundon CM Rellini AH Tonani S Santamaria V Nappi R. Mood disorders and sexual functioning in women with functional hypothalamic amenorrhea. Fertil Steril (2010) 94(6):2239–43. doi: 10.1016/j.fertnstert.2010.01.012 20206928

[34] Bomba M Corbetta F Bonini L Gambera A Tremolizzo L Neri F. Psychopathological traits of adolescents with functional hypothalamic amenorrhea: a comparison with anorexia nervosa. Eat Weight Disord (2014) 19(1):41–8. doi: 10.1007/s40519-013-0056-5 23912931

[35] Pentz I Nakić Radoš S. Functional hypothalamic amenorrhea and its psychological correlates: A controlled comparison. J Reprod Infant Psychol (2017) 35(2):137–49. doi: 10.1080/02646838.2016.1278201 29517358

[36] Tranoulis A Georgiou D Soldatou A Triantafyllidi V Loutradis D Michala L. Poor sleep and high anxiety levels in women with functional hypothalamic amenorrhoea: A wake-up call for physicians? Eur J Obstet Gynecol Reprod Biol X (2019) 3:1–5. doi: 10.1016/j.eurox.2019.100035 PMC6687383 31403123

[37] Tranoulis A Soldatou A Georgiou D Mavrogianni D Loutradis D Michala L. Adolescents and young women with functional hypothalamic amenorrhoea: is it time to move beyond the hormonal profile? Arch Gynecol Obstet (2020) 301(4):1095–101. doi: 10.1007/s00404-020-05499-1 32179966

[38] Strock NCA De Souza MJ Williams NI. Eating behaviours related to psychological stress are associated with functional hypothalamic amenorrhoea in exercising women. J Sports Sci (2020) 38(21):2396–406. doi: 10.1080/02640414.2020.1786297 32619140

[39] Bagby RM Parker JD Taylor GJ. The twenty-item Toronto alexithymia scale–I. Item selection cross-validation factor Struct J Psychosom Res (1994) 38(1):23–32. doi: 10.1016/0022-3999(94)90005-1 8126686

[40] Spielberger CD. State-trait anxiety inventory: bibliography. 2nd edn. Palo Alto: Consulting Psychologists Press (1989).

[41] Zung WW. A rating instrument for anxiety disorders. Psychosomatics (1971) 12:371–9. doi: 10.1016/S0033-3182(71)71479-0 5172928

[42] Dunstan DA Scott N. Norms for zung's self-rating anxiety scale. BMC Psychiatry (2020) 20(1):1–8. doi: 10.1186/s12888-019-2427-6 32111187 PMC7048044

[43] First MB Gibbon M Spitzer RL Williams JBW. User’s guide for the structured clinical interview for DSM-IV-TR axis I disorders - research version – (SCID-I for DSM-IV-TR, november 2002 revision). New York: Biometrics Research Department, New York State Psychiatric Institute (2002).

[44] Zung WW. A self-rating depression scale. Arch Gen Psychiatry (1965) 12:63–70. doi: 10.1001/archpsyc.1965.01720310065008 14221692

[45] Beck AT Ward CH Mendelson M Mock J Erbaugh J. An inventory for measuring depression. Arch Gen Psychiatry (1961) 4:561–71. doi: 10.1001/archpsyc.1961.01710120031004 13688369

[46] Kovacs M. The children's depression inventory (CDI). Acta Paedopsychiatrica: Int J Child Adolesc Psychiatry (1985) 21(4):995–8. doi: 10.1037/t00788-000 4089116

[47] Beck AT Steer RA Brown GK. Manual for the beck depression inventory-II. San Antonio, TX: Psychological Corporation (1996).

[48] Hamilton M. Development of a rating scale for primary depressive illness. Br J Soc Clin Psychol (1967) 6(4):278–96. doi: 10.1111/j.2044-8260.1967.tb00530.x 6080235

[49] Thelen MH Farmer J Wonderlich S Smith M. A revision of the bulimia test: The BULIT–r. Psychol Assess (1991) 3:119 –24. doi: 10.1037/1040-3590.3.1.119

[50] Weissman AN Beck AT. Development and validation of the dysfunctional attitude scale: a preliminary investigation. Annu Convention Am Psychol Associatio (1978).

[51] Garner DM Olmsted MP Bohr Y Garfinkel PE. The eating attitudes test: psychometric features and clinical correlates. Psychol Med (1982) 12(4):871–8. doi: 10.1017/s0033291700049163 6961471

[52] Cash T Henry P. Women’s body images: the results of a national survey in the USA. Sex Roles (1995) 33:19–28. doi: 10.1007/BF01547933

[53] Stunkard AJ Messick S. The three-factor eating questionnaire to measure dietary restraint, disinhibition and hunger. J Psychosom Res (1985) 29(1):71–83. doi: 10.1016/0022-3999(85)90010-8 3981480

[54] Garner DM Olmstead MP Polivy J. Development and validation of a multidimensional eating disorder inventory for anorexia nervosa and bulimia. Int J Eat Disord (1983) 2:15–34. doi: 10.1002/1098-108X(198321)2:2<15::AID-EAT2260020203>3.0.CO;2-6

[55] Patton GC Carlin JB Shao Q Hibbert ME Rosier M Selzer R. Adolescent dieting: Healthy weight control or borderline eating disorder? J Child Psychol Psychiatry (1997) 38(3):299–306. doi: 10.1111/j.1469-7610.1997.tb01514.x 9232476

[56] McNair D Lorr M Droppelman L. Manual for the profile of mood states. San Diego: Educational and Industrial Testing Service (1971).

[57] Frost RO Marten PA. Perfectionism and evaluative threat. Cogn Ther Res (1990) 14(6):559–72. doi: 10.1007/BF01173364

[58] Craig CL Marshall AL Sjöström M Bauman AE Booth ML Ainsworth BE. International physical activity questionnaire: 12-country reliability and validity. Med Sci Sports Exerc. (2003) 35(8):1381–95. doi: 10.1249/01.MSS.0000078924.61453.FB 12900694

[59] Papathanasiou G Georgoudis G Papandreou M Spyropoulos P Georgakopoulos D Kalfakakou V. Reliability measures of the short international physical activity questionnaire (IPAQ) in Greek young adults. Hellenic J Cardiol (2009) 50(4):283–94. doi: 10.1186/1471-2288-8-63 19622498

[60] Rosenbaum M. A schedule for assessing self-control behaviors: Preliminary findings. Behav Ther (1980) 11(1):109–21. doi: 10.1016/S0005-7894(80)80040-2

[61] McCoy NL. McCoy Female sexuality questionnaire. Qual Life Res (2000) 9:739–45. doi: 10.1023/A:1008925906947

[62] Smith BW Dalen J Wiggins K Tooley E Christopher P Bernard J. The brief resilience scale: assessing the ability to bounce back. Int J Behav Med (2008) 15(3):194–200. doi: 10.1080/10705500802222972 18696313

[63] Brantley PJ Waggoner CD Jones GN Rappaport NB. A daily stress inventory: development, reliability, and validity. J Behav Med (1987) 10(1):61–74. doi: 10.1007/BF00845128 3586002

[64] Cohen S Kamarck T Mermelstein R. A global measure of perceived stress. J Health Soc Behav (1983) 24(4):385–96. doi: 10.2307/2136404 6668417

[65] Perris C Jacobsson L Lindström H von Knorring L Perris H. Development of a new inventory assessing memories of parental rearing behaviour. Acta Psychiatr Scand (1980) 61(4):265–74. doi: 10.1111/j.1600-0447.1980.tb00581.x 7446184

[66] Soldatos CR Dikeos DG Paparrigopoulos TJ. Athens Insomnia scale: validation of an instrument based on ICD-10 criteria. J Psychosom Res (2000) 48(6):555–60. doi: 10.1016/s0022-3999(00)00095-7 11033374

[67] Mountjoy M Sundgot-Borgen J Burke L Ackerman KE Blauwet C Constantini N. International Olympic committee (IOC) consensus statement on relative energy deficiency in sport (RED-s): 2018 update. Int J Sport Nutr Exerc Metab (2018) 28(4):316–31. doi: 10.1123/ijsnem.2018-0136 29771168

[68] Berga SL Daniels TL Giles DE. Women with functional hypothalamic amenorrhea but not other forms of anovulation display amplified cortisol concentrations. Fertil Steril (1997) 67(6):1024–30. doi: 10.1016/s0015-0282(97)81434-3 9176439

[69] Lazarus RS Folkman S. Stress, appraisal, and coping. New York: Springer publishing company (1984).

[70] De Gucht V Heiser W. Alexithymia and somatisation: quantitative review of the literature. J Psychosom Res (2003) 54(5):425–34. doi: 10.1016/s0022-3999(02)00467-1 12726898

[71] Berga SL Marcus MD Loucks TL Hlastala S Ringham R Krohn MA. Recovery of ovarian activity in women with functional hypothalamic amenorrhea who were treated with cognitive behavior therapy. Fertil Steril (2003) 80(4):976–81. doi: 10.1016/s0015-0282(03)01124-5 14556820

