# Supplementary figures and images for: Psychological factors in functional hypothalamic amenorrhea: A systematic review and meta-analysis
Source: Front Endocrinol (Lausanne). 2023 Jan 27;14:981491. doi: 10.3389/fendo.2023.981491 (PMC9911452; doi:10.3389/fendo.2023.981491)

**Figure A. Funnel plot of the results for depression**

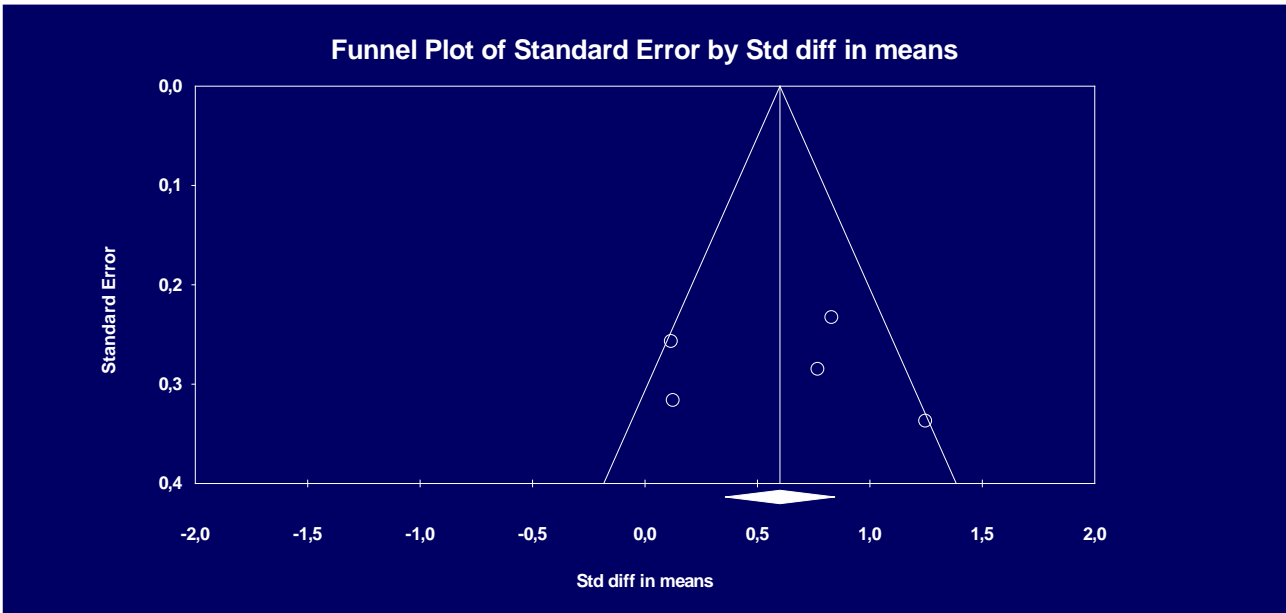

Supplement: Supplementary file 1 [file DataSheet_1.pdf]

Figure B. Funnel plot of the results for drive for thinness

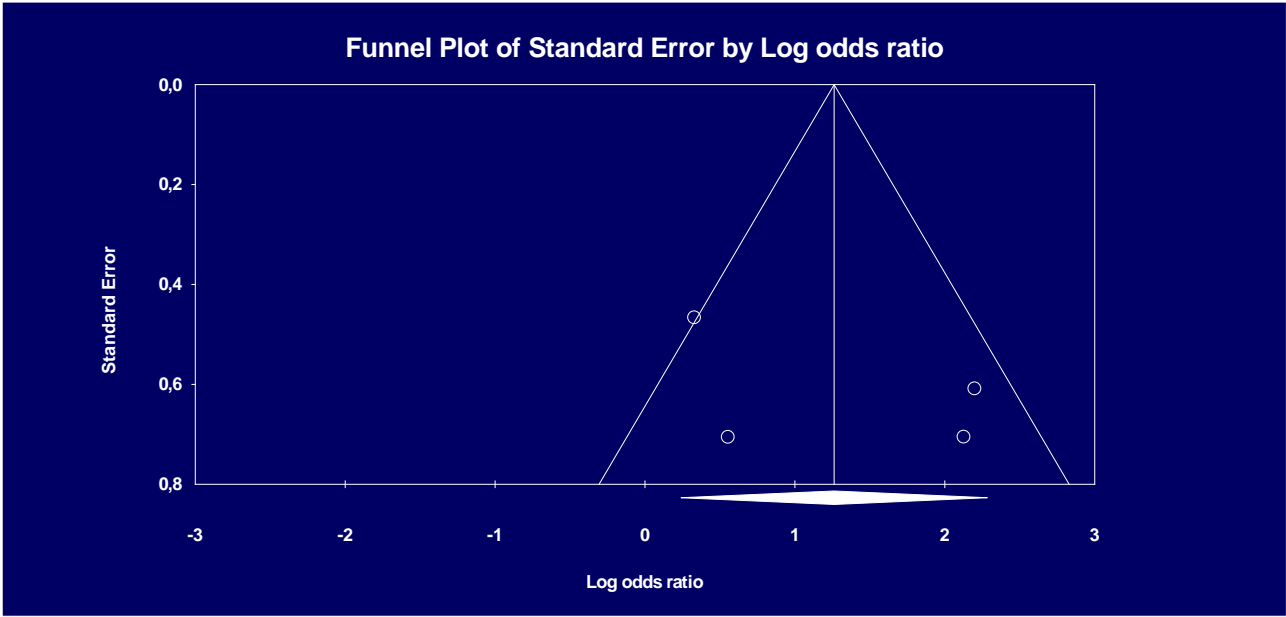

Supplement: Supplementary file 2 [file DataSheet_2.pdf]
