# Supplementary material for: Psychological factors in functional hypothalamic amenorrhea: A systematic review and meta-analysis
Source: Front Endocrinol (Lausanne). 2023 Jan 27;14:981491. doi: 10.3389/fendo.2023.981491 (PMC9911452; doi:10.3389/fendo.2023.981491)
Supplement: Supplementary file 3 [file Table_1.docx]

**Supplementary Table A_Risk of bias**

|  | Marcus et al. (2001) | Bomba et al. (2007) | Dundon et al. (2010) | Bomba et al. (2014) | Pentz & Radoš (2017) | Tranoulis et al. (2019-2020) | Strock et al. (2020) |
| --- | --- | --- | --- | --- | --- | --- | --- |
| 1.1 Is the source population or source area well described? | ++ | ++ | ++ | ++ | ++ | + | ++ |
| 1.2 Is the eligible population or area representative of the source  population or area? | ++ | ++ | + | ++ | ++ | - | ++ |
| 1.3 Do the selected participants or areas represent the eligible  population or area? | ++ | ++ | - | + | ++ | + | ++ |
| 2.1 Selection of exposure (and comparison) group. How was  selection bias minimised? | ++ | ++ | ++ | ++ | ++ | ++ | ++ |
| 2.2 Was the selection of explanatory variables based on a sound  theoretical basis? | ++ | ++ | ++ | ++ | ++ | ++ | ++ |
| 2.3 Was the contamination acceptably low? | ++ | ++ | ++ | ++ | ++ | ++ | ++ |
| 2.4 How well were likely confounding factors identified and  controlled? | ++ | ++ | ++ | ++ | ++ | ++ | ++ |
| 2.5 Is the setting applicable to the UK? | ++ | ++ | ++ | ++ | ++ | ++ | ++ |
| 3.1 Were the outcome measures and procedures reliable? | - | - | ++ | - | ++ | + | - |
| 3.2 Were the outcome measurements complete? | ++ | ++ | ++ | + | + | ++ | ++ |
| 3.3 Were all the important outcomes assessed? | ++ | ++ | ++ | ++ | ++ | ++ | ++ |
| 3.4 Was there a similar follow-up time in exposure and comparison  groups? | NA | NA | NA | NA | NA | NA | NA |
| 3.5 Was follow-up time meaningful? | NA | NA | NA | NA | NA | NA | NA |
| 4.1 Was the study sufficiently powered to detect an intervention  effect (if one exists)? | + | - | - | - | ++ | - | ++ |
| 4.2 Were multiple explanatory variables considered in the analyses? | ++ | ++ | ++ | ++ | ++ | ++ | ++ |
| 4.3 Were the analytical methods appropriate? | ++ | ++ | ++ | ++ | ++ | ++ | ++ |
| 4.4 Was the precision of association given or calculable? Is  association meaningful? | ++ | ++ | ++ | ++ | ++ | ++ | ++ |
| 5.1 Are the study results internally valid (i.e. unbiased)? | ++ | ++ | ++ | ++ | ++ | ++ | ++ |
| 5.2 Are the findings generalisable to the source population (i.e.  externally valid)? | ++ | ++ | ++ | ++ | ++ | ++ | ++ |
